# A Multi-Part Orientation Planning Schema for Fabrication of Non-Related Components Using Additive Manufacturing

**DOI:** 10.3390/mi13101777

**Published:** 2022-10-19

**Authors:** Osama Abdulhameed, Syed Hammad Mian, Khaja Moiduddin, Abdulrahman Al-Ahmari, Naveed Ahmed, Mohamed K. Aboudaif

**Affiliations:** 1 Industrial Engineering Department, College of Engineering and Architecture, Al-Yamamah University, Riyadh 11512, Saudi Arabia; 2 Raytheon Chair for Systems Engineering (RCSE Chair), Advanced Manufacturing Institute, King Saud University, Riyadh 11421, Saudi Arabia; 3 Advanced Manufacturing Institute, King Saud University, Riyadh 11421, Saudi Arabia

**Keywords:** additive manufacturing, build orientation, optimization, genetic algorithm, mass production

## Abstract

Additive manufacturing (AM) is a technique that progressively deposits material in layer-by-layer manner (or in additive fashion) for producing a three-dimensional (3D) object, starting from the computer-aided design (CAD) model. This approach allows for the printing of complicated shaped objects and is quickly gaining traction in the aerospace, medical implant, jewelry, footwear, automotive, and fashion industries. AM, which was formerly used for single part customization, is currently being considered for mass customization of parts because of its positive impacts. However, part quality and build time are two main impediments to the deployment of AM for mass production. The optimal part orientation is fundamental for maximizing the part’s quality as well as being critical for reducing the fabrication time. This research provides a new method for multi-part AM production that improves quality while reducing overall build time. The automatic setup planning or orientation approach described in this paper employs two objective functions: the quality of the build component and the build time. To tackle the given problem, it introduces a three-step genetic algorithm (GA)-based solution. A feature-based technique is utilized to generate a collection of finite alternative orientations for each component within a specific part group to ensure each part’s individual build quality. Then, a GA was utilized to find the best combination of part build orientations at a global optimal level to reduce material consumption and build time. A case study of orienting nine components concurrently inside a given building chamber was provided for illustration. The findings suggest that the developed technique can increase quality, reduce support waste, and shorten overall production time. When components are positioned optimally rather than in random orientations, build time and support volume are reduced by approximately 7% and 16%, respectively.

## 1. Introduction

Additive manufacturing (AM), in contrast to subtractive manufacturing, is a method of building three-dimensional (3D) objects by adding material in a layer-by-layer manner [1]. Its deployment has a lot of possibilities for high-performance component design. Implementing AM technologies can bring numerous advantages, including the ability to make components of any shape or functionality, reducing design difficulties, investment costs, and production time, as well as allowing for reconsidering product development rules [2,3,4]. Materials for AM have seen significant expansion in recent years, both in terms of utilizing new types of materials and in terms of sales volume [5,6,7]. Plastic, resin, rubber, ceramics, glass, concrete, metal, bio-inks, composites, yarn, and other materials can all be used in AM. The defense, energy, aerospace, automotive, and biomedical industries, among others, have all shown a strong interest in AM technologies [8]. Furthermore, AM, which was primarily used for prototyping, is now being used for mass customization in production. However, part quality and build time are two major roadblocks to the adoption of AM in mass production [9,10]. According to [11], the AM technique can produce many parts in the same machine at the same time. Thus, it can also be regarded as a serious and perfect technology for multiple component manufacturing.

The quality of the manufactured component and the time it takes to build it are the most important considerations in AM [12]. The fundamental quality constraints of AM parts are poor surface finish and dimensional and geometrical inaccuracies [13]. A variety of rules, including horizontal and perpendicular faces in the build direction, cylindrical or round features aligned with the *z*-axis, count of curved surfaces in the horizontal plane, base surface area, count of angular and inclined surfaces, overhanging area, trapped volume, and so on, can influence the quality of AM parts. Similarly, fabrication time is an important factor to consider. Machine variables, part shape, part height, layer thickness, support volume, etc., all have an impact on the AM process. It is also worth noting that quality and build time are inextricably linked and must be examined together. For example, while a reduction in layer thickness may improve surface quality, it also increases the build time. Post-processing (removal of support structures) adds to the build time and expenses, and degrades the geometric quality [14]. An AM product, therefore, should not only meet quality standards, but also be made at the lowest possible cost and shortest time achievable [15]. The flexibility to establish appropriate component orientation is critical in AM, since it can increase part quality while also reducing build time [16,17,18]. The build orientation of a particular component influences the staircase repercussions, the support quantity, the support size, the number of supports required, the build time, the surface quality, process planning, post-processing, and economics [19]. 

Optimizing orientation for several parts in a single build has become extremely important because of the transition in AM applications from singular customization to mass customization. Owing to the complexity of acquiring suitable orientations for multiple parts, most earlier efforts have focused on single component optimization. This research, on the other hand, proposes a unique multi-part AM production approach that improves part quality while reducing total fabrication time. Since this is a multi-objective optimization problem, one criterion, such as part quality, is chosen as the primary goal, with the other serving as a secondary objective. This strategy is more straightforward and more in line with the actual needs of the user [15]. The automatic setup planning or orientation methodology discussed in this paper uses two objective functions. The primary objective function improves the quality and accuracy of the built component, while the secondary objective function reduces build time. The technique begins with data extraction from a Standard for the Exchange of Product model data (STEP) AP 203 E2 file, followed by autonomous slicing using a CATVBA file within CATIA V5. The obtained feature and slicing data serve as the foundation for optimum setup design. It uses a genetic algorithm (GA)-based meta-heuristic optimization to obtain the proper orientation while simultaneously enhancing the quality and reducing build time for multiple components.

## 2. Literature Survey

The build orientations of manufactured components influence the staircase adverse effect, the support density, the support area, the degree of supports required, the build time, the surface quality, mechanical strength, process design, postprocessing, and budget [19,20,21,22,23]. Several efforts by various authors were published with the goal of finding the ideal build orientation of a 3D model in order to streamline the AM process [24,25,26,27,28,29,30]. 

The work reported in [31] focused on restricting the lower bound of the overhang angle in AM to eradicate or lessen support structures. Similarly, a novel statistical method was applied to establish a rapid automatic decision support system for identifying suitable AM build orientation [32]. A fuzzy multi-attribute decision-making approach was developed as a useful tool for acquiring build orientations automatically [28]. Alternative build orientations and ideal build orientations were the two primary components in the process. This method produced a score of all possible build orientations as a final outcome. Part build orientation is a critical process parameter that influences part quality, namely Geometric Dimensioning and Tolerancing (GD&T) errors, energy consumption, and the amount of support structures required. Das et al. [33] devised an approach to find out an optimal build orientation that decreased the volume of support structures while achieving the part’s stated GD&T parameters for a Direct Metal Laser Sintering (DMLS)-based process. The optimization model leveraged the Siemens PLM NX API, a Quadtree decomposition, and mathematical correlations to find the ideal build orientations for lowering support structures while satisfying design specifications. A feature-based design technique was used to facilitate the build orientation evaluation [34]. The approach used a ray-tracing and convex hull method to find possible build orientations following automated part tessellation and detection of outside part surfaces. Alternatives were graded based on their ability to reduce overhang structures while simultaneously reducing the demand for additional support structures. 

The implications of the stair-stepping effect are greatly influenced by a surface’s build angle, while the aggregate surface quality is also affected by the resolution of the individual AM process. Delfs et al. [35] evaluated the surface quality of a part based on its build orientation. Since not every portion of a component’s surface is evenly essential, a preselection of areas was used to strengthen the overall surface quality of key regions. The developed model made use of a part’s digital Additive manufacturing file format (AMF). Each triangle was allocated a roughness value, and the ideal one was determined by experimenting with various orientations. This method necessitated the creation of a surface quality database, and it was conducted independently for each AM process. The developed technique generated outstanding results using a surface topography simulation for the laser sintering process as an example. Jaiswal et al. [36] developed a novel method for determining the best build orientation for functionally graded material (FGM) artifacts. They designed a cost function for build orientation optimization (BOO) that included material and geometric errors as major considerations. The geometric error was used to address failures generated by the discretization of material composition along the toolpath’s cross-section, while the material error was used to manage inadequacies induced by volumetric staircase errors. A surrogate model-based global optimization was built to tackle the stated BOO problem, and the proposed optimization framework was evaluated using different sample artifacts to demonstrate the entire technique and show its usefulness. A new multi-criteria optimization formulation that used meta-modeling, Non-Dominated Sorting Genetic Algorithm–II (NSGA II), and Technique for Order Performance by Similarity to Ideal Solution (TOPSIS) was proposed by Khodaygan and Golmohammadi [37] to discover the exact optimum build orientation in the AM. The build time and surface roughness as objective functions were defined in explicit form in terms of part orientation, using the Kriging method as a powerful meta-modeling tool. To determine the Pareto-optimal solutions, the NSGA-II was used. The TOPSIS approach was used to score all of the generated optimum solutions to find the optimal solution. A cutting-edge optimization solver was used by Pereira et al. [38] to manage the optimization of the AM-produced surface. It addressed the constraints imposed by the model orientation, support formation, and slicing processing steps, with the goal of reducing the staircase consequence and the necessity for supports. Part orientation and support generation are linked because the optimal orientation of the component to be produced reduces production time and lowers the necessity for support generation, resulting in improved surface quality. Slicing is the process of dividing an item into layers, with the staircase effect posing a particular challenge for artifacts with steep slopes and curvatures, causing higher surface roughness. The solvers based on global derivative-free optimization restricted to reasonable limits on the variables were chosen to accomplish the objectives [39,40]. GA, due to its capacity to uncover global optimum and avoid trapping in local optima, was also exploited by Luo et al. [20] to optimize the build orientation by examining the least volume of the support structure and the minimal strain energy of a part. A single weighted sum function (WSF) was established to minimize the volume of the support structure and the strain energy at the same time. There have been various studies in the past, as shown in Table 1, that highlighted the importance of using optimization strategies to establish the ideal build orientation for AM. The fundamental concept of optimization is to find a build orientation that allows for the optimization of one or more relevant goals, including design specifications, manufacture price, production time, part quality, mechanical characteristics, thermodynamic properties, and so on.

Many scholars have looked at the subject of built orientation optimization and presented a variety of solutions. Past studies, on the other hand, have primarily focused on determining an ideal orientation for a single component, with few solutions for the multi-part production orientation optimization problem, in which a group of components (in the single build) should be optimally orientated at the same instant. For example, Jiang et al. [70] concocted a four-step multi-part production strategy for decreasing AM support usage. The suggested method integrated precisely two components based on their geometries, identified a number of potential multi-part combinations, and then chose the ideal part placements for production. Similarly, an eight-step innovative technique for multi-part AM was suggested by Jiang et al. [71] in order to shorten overall manufacturing time and acquire optimal part locations and nozzle travel paths. In order to decrease the overall manufacturing cost and time, studies including part-to-printer allocation and stacking of multiple parts for a distributed network of Fused Deposition Modeling (FDM) were also conducted [72]. Ransikarbum et al. [73] optimized component orientation, part-to-printer allocation, and path planning with FDM 3D printers using an unique framework based on reasoning algorithms. Undoubtedly, efforts are being made to maximize the capabilities of 3D printers for multi-part production. Nevertheless, there is a significant gap in determining the appropriate build orientation for multiple parts in a single AM build. As a result, the primary goal of this study is to present a solution to the problem of multi-part orientation optimization. The suggested strategy generates a set of finite alternate optimal orientations for each individual part using a rule-based mechanism. After that, a GA was used to ascertain the optimal configuration of part build orientations in order to reduce build time and material consumption. The components are also manufactured at optimal orientation in the FDM machine to demonstrate the applicability of the developed approach.

Both acrylonitrile butadiene styrene (ABS) and polylactic acid (PLA) are the two most important and widely used materials for FDM 3D printing. However, ABS is preferred to PLA in this work due to the reasons listed below.

- ABS is typically preferred by engineers as the material of choice for industrial applications due to its enhanced ductility, high flexural strength, and greater elongation than PLA [74].
- Compared to PLA, ABS is more resistant to physical stress (UV radiation and high temperatures) [75].
- In addition to being stronger and lighter than PLA, ABS also absorbs moisture more slowly [74,76].
- PLA starts to lose its structural integrity and distort as it gets closer to 60 degrees, especially while carrying a larger load [77].
- PLA components should not be used outside more frequently because they deform quickly and soften when exposed to sunlight [78].

Since mostly mechanical or industrial parts have been considered in this work, therefore ABS is preferred due to its superior qualities in regard to ductility, strength, thermal stability, flexural strength, light-weight, sustainability, durability, functionality and end-use industrial applications.

## 3. Methodology

The working principle of AM approach is dependent on layer-by-layer printing, in which each layer is placed on another layer for fabrication [70]. Support structures are typically generated to help with overhanging features, holes, or edges. The fundamental objective of the support structures is to stabilize the component and provide protection against gravitational forces. Print orientation is a key aspect in AM since it affects support usage, build time, and the overall manufactured qualities [79]. Therefore, in this work, a GA-based optimization algorithm was developed to minimize support volume, build time, and maximize part’s quality or accuracy. 

The primary step in this approach is the description of an objective function which is defined using Equation (1) for the problem presented in this work. It is based on two criteria, *Q* and *T*, as shown in Equation (1).

(1) $$\mathrm{Min}\;Z=\left(\frac{1}{Q}\right)+T$$

Prior to the minimization of *Z* using GA, *Q* and *T* have to be computed. Since it is a multi-part orientation problem, the values of *Q* and *T* have to be computed for every orientation of each part. The procedure for estimating *Q* and *T* is discussed below.

### 3.1. Estimation of the First Criterion (Q)

The primary criterion (*Q*) enhances the quality/accuracy of the building part. It relies on the optimization of GD&T for all part faces. It suggests that maximum GD&T can be achieved by the maximization and minimization of the following factors. It proposes that maximization and minimization of the relevant criteria can lead to maximum GD&T.

1. Parallel and perpendicular surfaces must increase along the z-direction (build axis).
2. Cylindrical structures (a hole, cone, etc.) with their axis aligned in the z-direction should be maximized.
3. The count of curved surfaces must be higher in the horizontal plane.
4. The base surface area has to be maximized.
5. Angular/inclined surfaces should be minimized.
6. The overhanging area must be reduced.
7. Trapped volume has to be minimum.

The approach used to estimate the first criterion (*Q*) is described below. The primary criterion emphasizes the part’s quality and accuracy. As a result, the largest value of this criterion is used to determine its optimal orientation. For all part faces, the greatest GD&T is used to get the maximum value. A decrease of inclination and support structure regions achieves maximum GD&T for the complete part spanning different face types (straight, inclined, and cylindrical). In AM techniques, the inclined portion creates challenging staircase consequences. Weight parameters were established for all face types present in the component to lessen the staircase issue and support structure region [15,41,62].

$$\begin{array}{l} w_{1j}=\left\{\begin{array}{cc} 0 & \theta_{j}=\left\{0^{{}^{\circ}},\;90^{{}^{\circ}},\;180^{{}^{\circ}}\right\}\\ -\cos\frac{\theta }{2} & otherwise, \end{array}\right.\\ w_{2j}=\left\{\begin{array}{cc} 0 & no\;support\\ -0.3 & need\;support \end{array}\right.\\ w_{3j}=\left\{\begin{array}{cc} 0 & no\;overcure\\ -0.2 & need\;overcure \end{array}\right.\\ W_{j}=\left\{\begin{array}{cc} 1+w_{1j}+w_{2j}+w_{3j} & \begin{array}{cc} \theta_{j}= & \left\{\begin{array}{lll} 0^{{}^{\circ}},\; & 90^{{}^{\circ}},\; & 180^{{}^{\circ}} \end{array}\right\} \end{array}\\ w_{1j}+w_{2j}+w_{3j} & \theta_{j}=]\begin{array}{lll} 0^{{}^{\circ}},\; & 90^{{}^{\circ}}\;\left[U\right]\;90^{{}^{\circ}}, & 180^{{}^{\circ}} \end{array}[\\ -1+w_{1j} & w_{1j}\neq w_{2j}\neq w_{3j}\;\neq 0 \end{array}\right. \end{array}$$

(2) $$Q_{i}=\sum_{j=1}^{n}N_{ij}W_{j}$$

(3) $$N_{j}=\frac{n\;*\;A_{j}}{A_{total}}$$

where

*w*_1j,_*w*_2*j*_, *w*_3*j*_ = weight factors for inclined faces, supporting structures, and overcure surfaces, respectively.

$Q_{i}$: Objective value of the built part’s *i*th orientation

$N_{j}$: Relative count of areas of face *j*

$W_{j}$: Weights allocated to each face type

*n*: Maximum number of face types that can be used in a given orientation

$A_{j}$: Area of face

*A_total_*: Total face area of the component

*i*: Component’s *i*th orientation 

*j*: *j*th type of the component’s surface 

*θ*: Slope angle 

The value of *Q* is computed for each orientation, and finally, the maximum value is selected to be used in Equation (1). As a result, increasing the regions of perpendicular surfaces, the portions of upward-facing horizontal surfaces, the count of holes with their axes in the slicing direction, and the base surface area, while reducing the area of sloped surfaces, the total area of overhanging surfaces, and the area of trapped volumes, yields the highest GD&T. 

### 3.2. Estimation of Second Criterion (T)

The build time (Equation (1)) is the second significant criterion, especially when the first criterion has the same value for different part orientations (*Q_i_*). The minimum build time, which specifies a minimal number of slices and the least volumetric inaccuracy, is also estimated for alternative orientations. The length of time it takes to produce a component is determined by machine variables, part geometry, part height, layer thickness, and the quantity of support volume required. As a result, the build time varies depending on the part’s orientation. 

The time to prepare the Standard Tessellation Language (STL) file, machine setup time, component manufacturing time, and post-processing time are the key elements in computing overall building time. The total build time (*T_B_*) is derived by adding the time required to encompass the part geometry (*T_g_*), the time involved in generating support volume (*T_s_*), and the time utilized for machine movement, including the platform movement time (*T_m_*) [62].

(4) $$T_{B}=T_{g}+T_{s}+T_{m}$$

(5) $$T_{g}=\frac{Part\;Volume}{Building\;rate\;of\;machine}$$

(6) $$T_{s}=\frac{Support\;Volume}{Building\;rate\;of\;machine}$$

(7) $$T_{m}=T_{1}+T_{2}+T_{3}+T_{4}+T_{5}+T_{6}+T_{7}+T_{8}+T_{9}+T_{10}+T_{11}+T_{12}$$

where, *T*_1_ = Dip lag; *T*_2_ = Platform displacement time; *T*_3_ = Sweep cycle time; *T*_4_ = Z-hold up time; *T*_5_ = Tip purging time; *T*_6_ = Roller displacement time; *T*_7_ = Part-bed displacement time; *T*_8_ = Chamber displacement time; *T*_9_ = System lag; *T*_10_ = Over curing period; *T*_11_ = Warm up period; *T*_12_ = Cooling period

**Figure 1:**
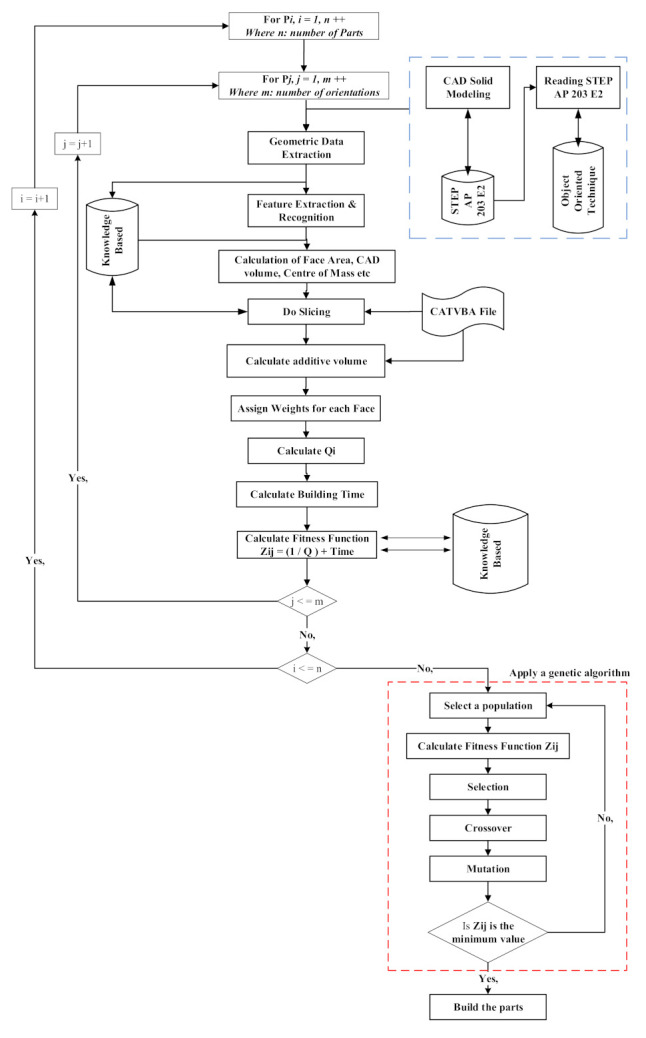
Methodology adopted to optimize orientation for multi-part production.

Figure 1 depicts the complete methodology adopted in this study. Data were extracted from a STEP AP 203 E2 file to initiate the approach. The data extraction output file was prepared by employing an object-oriented technique to retrieve the essential data from the STEP file. The feature faces were recognized and grouped, and geometric features such as parallelism, perpendicularity, and inclination were determined using C++ algorithms [80]. At this point, the face area, component volume, and center of mass were computed. Following that, the part’s technological properties are retrieved and recognized utilizing appropriate manufacturing guidelines [62,81]. The subsequent phase is to use a CATVBA file inside CATIA V5 to perform automatic slicing. The 3D component design is utilized as the source file for automatic slicing, while the part height and layer thickness are the slicing code’s inputs. Following that, the layer area and volume variables were automatically estimated, and the data were saved to an Excel datasheet. The overall additive volume of the component is computed using the exported data.

### 3.3. Optimization Using Genetic Algorithm

The objective function (Equation (1)) formulated using the theoretical model for the first criterion (Equations (2) and (3)) and additive model for the second criterion (Equations (4)–(7)) is too complicated, due to the presence of multiple orientations for multiple parts, to be evaluated utilizing any standard optimization technique. GA is a powerful tool for optimizing such objective functions. The use of GA is motivated by the fact that it is a straightforward and effective optimization tool for solving NP-hard problems. It optimizes using a set of solutions (known as population size) and returns a set of sub-optimal solutions, as opposed to standard optimization methods, which begin with just one feasible solution and produce only one optimum response, which might or might not be the global optimum solution. To determine the ideal solution, GA employs three basic evolutionary operators: selection, crossover, and mutation. The selection operator chooses candidate chromosomes from the entire population, which are then subjected to crossover and mutation modifications. By recombination from the chosen parent chromosomes, the crossover operator generates fresh offspring. For the purpose of preventing early convergence, the mutation operator modifies the gene of chromosomes arbitrarily. The following are the primary steps in establishing the GA [59].

**Figure 2:**
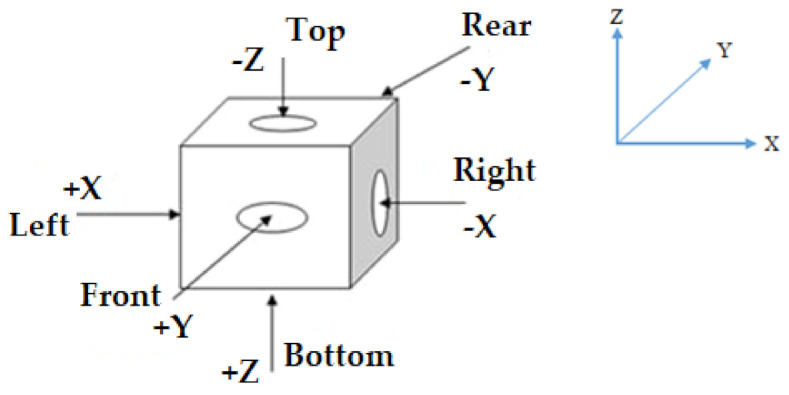
Designation of component’s faces.

**Encoding**: Individuals (chromosomes) in GA-based algorithms are made up of a set of genes that can be expressed as integers, Boolean variables, string variables, etc. One of the most important processes in GA is chromosomal encoding, which is largely dependent on the structure of the problem to be addressed. In this work, permutation encoding has been selected, where every orientation (chromosome) is symbolized by a string of numbers, such as 1 4 6 2 5 8, and so on [82]. For illustration, an orientation arrangement for a problem with nine parts can be defined using a chromosome, *C* = [2 4 1 3 2 6], which means that the orientation of the first part is 2, the orientation of part 2 is 4, the orientation of part 3 is 1, and so on. The different orientations are explained in Table 2. Before implementing GA, the faces in all parts are defined as bottom, top, front, rear, right, and left, as shown in Figure 2. To create a newer group of highly competent individuals, successive processes in this approach necessitate the use of evolutionary operators like selection, crossover, and mutation.

**Initialization:** Random initial solutions are triggered during the initialization stage, and the objective function score for these alternatives is determined. Random population initialization is the basic and frequent population generation strategy where initial solutions are chosen randomly [83]. A random number between 1 and *‘population size’* is estimated during the creation of every individual; if the derived number is already in the existing individual, a fresh number is generated; otherwise, the generated number is concatenated to the current individual and the process is iterated until the individual’s length attains the predetermined value of population size.

**Selection**: The roulette selection mechanism is a prominent and convenient option for a single-objective optimization problem [59]. The two-objective optimization problem is condensed to a singular optimization problem in this work. As a result, the roulette selection operator is utilized for the selection process, which is extensively employed in single-objective optimization problems. It is based on the philosophy of selecting the best individuals and leaving the rest of the individuals. The individual with the best fitness value has the highest probability of being selected as a parent for subsequent operations, and its convergence rate is fast. The algorithm for roulette wheel selection can be described as follows [84].

**STEP 1:** Compute the fitness function values (*f*) depending on *Z* values.

**STEP 2**: Compute *p_i_* = $\frac{f_{i}}{\sum_{i=1}^{N_{P}}f_{i}}$, ∀*i* = 1, 2, 3, 4, …, *N_P_*. Where *pi* is the probability of each chromosome to be a parent in the next operation, *N_P_* is the population size.

**STEP 3:** Estimate the cumulative probability for each of the chromosomes starting from the top of the list, i.e., *P_i_* = $\sum_{j=1}^{i}p_{j}$, ∀*j* = 1, 2, 3, 4, …, *N_P_*. Where *Pi* is the cumulative probability of each chromosome.

**STEP 4**: Generate a random number, “*rand*” between 0 and 1.

**STEP 5**: Select the *i*th individual such that *P_i-1_* < *rand* < *P_i_*.

**STEP 6**: Repeat Steps 4 and 5 to select *N_p_* individuals. (Assuming population size = mating pool size).

End

Table 3 lists a sample population of three chromosomes (a typical population of 50 would be difficult to illustrate).

As a result, the roulette wheel selection procedure was applied to pick the chromosomes I and II for reproduction, and they then underwent a crossover operation. STEPS 5 and 6 will be repeated until the requisite number of individuals has been obtained.

**Figure 3:**
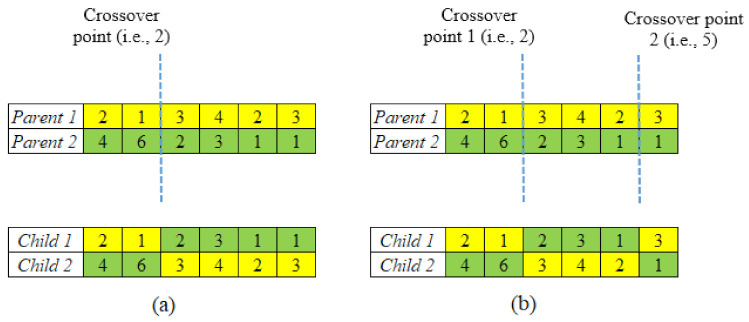
Operation of (**a**) single-point crossover; (**b**) two-point crossover.

**Crossover**: A crossover operation was implemented to promote population variety that facilitates global searching. One of the most difficult aspects of using evolutionary algorithms, such as GA, to solve combinatorial optimization problems is designing an effective crossover operator. Since the chromosomes have been characterized by a string of numbers using the permutation encoding technique, and the repetition of genes (as multiple parts can have the same orientation) is permissible, the two-point crossover is used in this study. Two-point crossover has various advantages because of its convenience, flexibility, and expediency [85]. The functioning of the single and two-point crossovers can be seen in Figure 3 [86,87].

An arbitrary crossover location was chosen in this one-point crossover, and the ends of its two parents were exchanged to produce new offspring [85,88]. Two-point crossover, which is an extension of single-point crossover, involves the swapping of alternating segments to get new off-spring. The crossover procedure for a pairing of chromosomes containing six genes is as follows.

**STEP 1:** Randomly acquire two parents from the “Selection” pool.

**STEP 2**: Generate two random numbers, *rand*, and *rand’* (1 < *rand* (and *rand’*) < *n* as well as *rand < rand´*; *n* is the number of genes in the chromosome) that designate two crossover points. In the given example (Figure 3), two randomly generated crossover points are 2 and 5.

**STEP 3**: The genes (or orientations) at *i* = 1, 2, …, *rand* and *i* = *rand’*, *rand’*+1, …, *n*, in *Child 1* are taken from Parent 1, while genes at *i* = *rand*, *rand*+1, …, *rand’*, are taken from Parent 2.

**STEP 4**: The genes (or orientations) at *i* = 1, 2, …, *rand* and *i* = *rand’*, *rand’*+1, …, *n*, in *Child 2* are taken from Parent 2, while genes at *i* = *rand*, *rand*+1, …, *rand’*, are taken from Parent 1.

Repeat STEPS 1–4 until the desired number of individuals are obtained (in the present case, it is equal to the population size).

**Figure 4:**
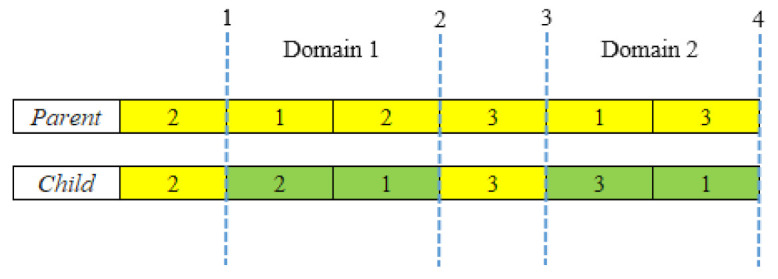
Working of double-inversion mutation operator [89].

**Mutation**: The mutation operation is exploited to maximize population heterogeneity when the optimization mechanism nears ‘mature’. This operation in GA can accelerate local search while also preventing ‘premature’ results. The genes in this instance occupy fixed locations in a chromosome. It is worth noting that the crossover aids population convergence by homogenizing chromosomes, but the mutation, in the case of the local optimum, restores genetic variation to the population. The double inversion-based mutation operator employed in this study is inspired by the work of Xin et al. [89]. The inversion type mutation operator is efficient and greatly minimizes the probability of trapping in local minima [89,90]. The double inversion mutation procedure is described in Figure 4. The inversion mutation operator flips the arbitrarily picked substring across any two designated positions in an individual [85,91]. The double inversion procedure for a chromosome with six genes can be described as follows.

**STEP 1:** Randomly acquire a chromosome from a population.

**STEP 2**: Generate four random numbers, *rand*, *rand’*, *rand1*, and *rand1’*, such that

1 < rand (rand’, rand1, and rand1’) < n and

*rand < rand’* < *rand1* < *rand1’* (*n* is the number of genes in the chromosome)

The region between *rand* and *rand’* represents Domain 1, while the region between *rand1* and *rand1’* represents Domain 2. 

**STEP 3**: The segments in both domains are reverted at the same time to yield a child chromosome.

**STEP 4**: The fitness of both the child and the parent chromosomes is assessed in order to establish which one is better for the upcoming generations.

Repeat STEPS 1–4 until the desired number of individuals is obtained. With the double-domain inversion, it is possible to guarantee the evolution towards a global optimum.

**Termination**. When the count of produced individuals in the population meets the preset level, the process ends. As a result, the program ends after a certain count of iterations, i.e., when the total count of iterations in the problem hits the maximal count of iterations. This criterion is chosen since it guarantees outcomes in a reasonable amount of time, regardless of whether the algorithm achieves higher levels or not [82]. Furthermore, this criterion has resulted in a reduction in Central Processing Unit (CPU) time. A fresh search can be initiated if no acceptable solution is reached.

The pseudocode, or the framework for applying a GA-based algorithm, is given below.

Select GA parameters (population size, crossover probability, mutation probability, and maximum number of generations)

BEGIN
Encoding of chromosome (Permutation Encoding)
Population Size
Crossover Probability
Mutation Probability
Generate initial solutions randomly with *N_p_* population size
Evaluation of each individual in the population based on objection function
WHILE (Generation < Termination Criteria)
Selection (Roulette Selection)
Crossover (Two-point crossover)
Mutation (Double inversion mutation)
Evaluation of individuals in the new population
Select the best solutions to become the next generation
Generation = Generation+ 1
REPEAT
END WHILE
Retain the best solution obtained.

## 4. Case Study

**Figure 5:**
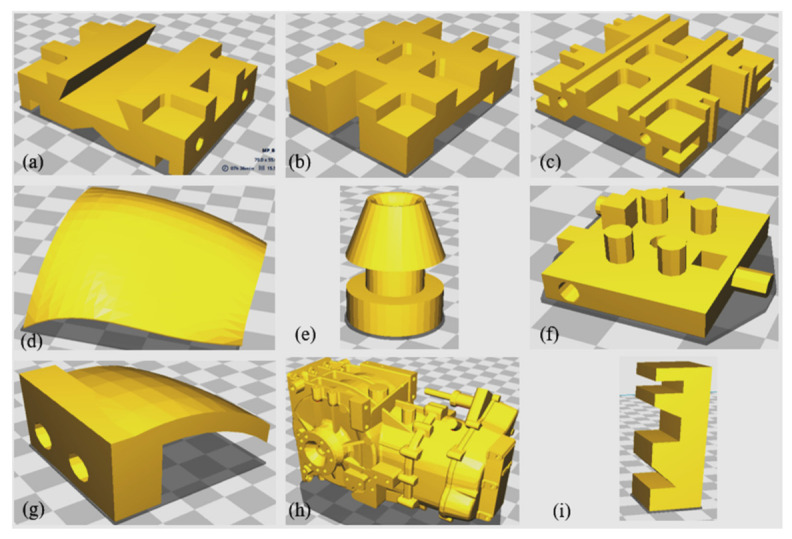
Case Studies (**a**) Component 1; (**b**) Component 2; (**c**) Component 3; (**d**) Component 4; (e) Component 5; (**f**) Component 6; (**g**) Component 7; (**h**) Component 8; (**i**) Component 9.

**Figure 6:**
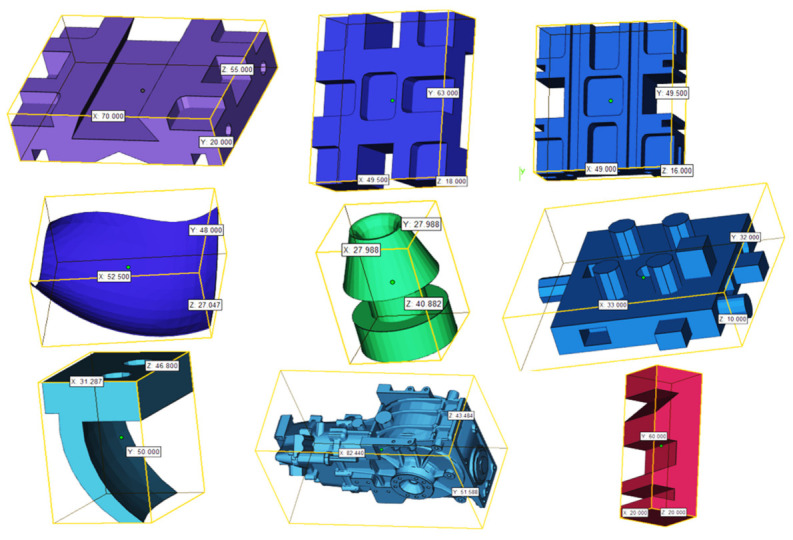
Components and their dimensions.

The proposed methodology is demonstrated through a case study that includes the fabrication of nine components with six different orientations using an FDM machine. The nine components vary in size, complexity, number of features, and application. CATIA V5 is used to construct the 3D solid models for these components, which are illustrated in Figure 5. An object-oriented technique was used to extract geometric and topological information from the STEP AP 203 file. Table 4 shows the details of each of these components. For example, fifty-seven faces, along with their loops, edges, vertices, face orientation, face type, and face area, were retrieved from a geometric information file for component 1. Fourteen manufacturing features, as well as their dimensions, were also extracted and recognized. The dimensions of each of the components are shown in Figure 6.

**Figure 7:**
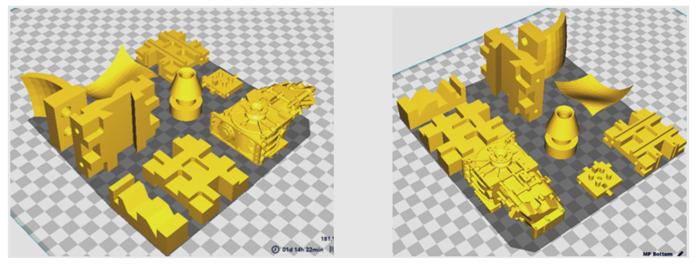
Components in two different random orientations on the build platform.

**Figure 8:**
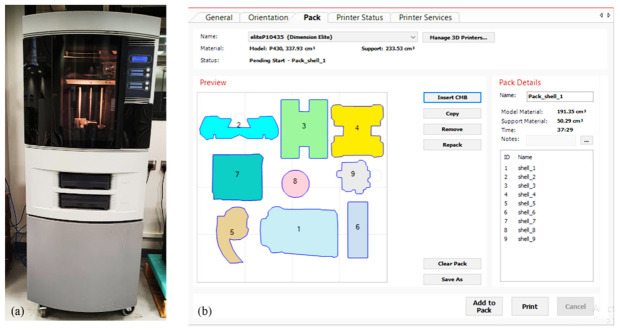
(**a**) Dimension Elite 3D printer (Courtesy: STRATASYS); (**b**) Component arrangement.

The components can be arranged in any combination of orientations (as illustrated in Figure 7) as long as the overall dimensions of the components indicated above are within the build envelop of the machine. Their random orientation, on the other hand, would result in more material consumption (due to additional supports), longer build times, and lower accuracy in FDM. As a result, it is vital to arrange the components in the appropriate orientations. The Dimension Elite 3D printer (Courtesy: STRATASYS) was used to produce the components, as illustrated in Figure 8a. It uses ABS as a material for part fabrication and is based on the FDM technique. This machine can produce parts up to 203 × 203 × 305 mm in size, with layer thicknesses ranging from 0.178 mm to 0.254 mm. The components as depicted in Figure 8b are packed such that they are well within the build envelop or build table.

Before implementing GA, it is important to compute *Q* (first criterion) and *T* (second criterion) for each orientation of every component using Equations (2) and (3) and Equations (4)–(7), respectively. The outcome of these values is depicted in Table 5.

**Figure 9:**
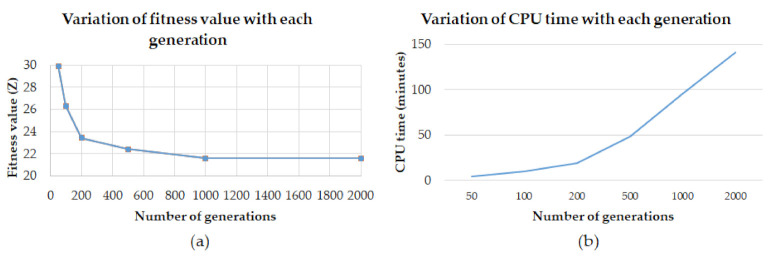
(**a**) Change in fitness value; (**b**) Variation in CPU time.

The next step is the implementation of GA for identifying the optimum orientation of components on the build table for AM. The foremost step in the implementation of GA is the selection of GA parameters. The parameters of GA employed are: population size: 50, generations = 2000, crossover-type: Two-point, crossover probability: 0.85, mutation type: Double inversion, mutation probability: 0.05. The population size is decided by experiments. Multiple population sizes from 10 to 50 were explored, and it was ascertained that beyond population size 50, there was no notable difference in solutions, as well as after 2000 generations, there is no considerable variation in observations (see Figure 9a). A literature survey was used to obtain appropriate crossover and mutation probabilities. It is generally considered that crossover has a higher probability, usually, 0.8–0.95 and mutation probability is generally low, from 0.001 to 0.05 [92]. The procedure is carried out utilizing the MATLAB application on a machine with Intel(R) Core (TM) i7-5600U CPU running at 2.60 GHz 8 GB RAM. Outcomes in terms of elapsed time for program running (see Figure 9b) and *Z* value (Equation (1)) obtained for various orientations of nine parts were collected.

**Figure 10:**
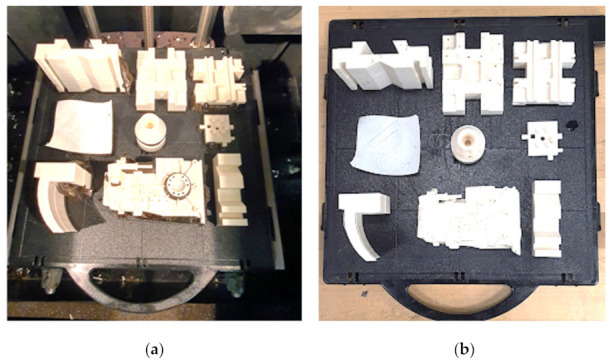
Fabricated components manufactured using FDM. (**a**) With supports; (**b**) After removing supports.

The parts were built in the FDM machine once the optimum orientation was determined using GA, and the manufactured parts are displayed in Figure 10a,b. 

**Figure 11:**
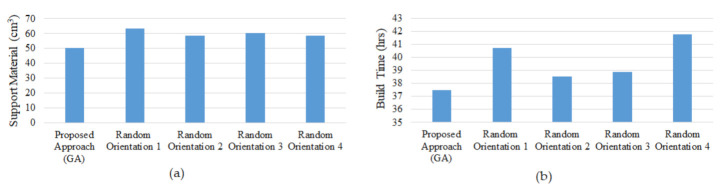
Comparison of optimum and random orientations (**a**) Support Material; (**b**) Build Time.

The fabrication of nine components at their optimal orientations requires about 38 h and 50.29 cm^3^ of support material as opposed to 40 h and 60.27 cm^3^ of support material when they are built arbitrarily. Figure 11a,b compares having the various parts positioned in an optimal manner with having them oriented randomly. It demonstrates that components produced in the optimal orientation perform better in terms of support material and build time. It reveals that by placing components optimally rather than randomly, support material and build time can be reduced by at least 16.57% and 6.68%, respectively. A 1 cm^3^ of support material costs around $ 0.34, therefore the total cost of building in optimal orientation would be around $17 versus $21 for random orientations, saving about $4 on each build. 

**Figure 12:**
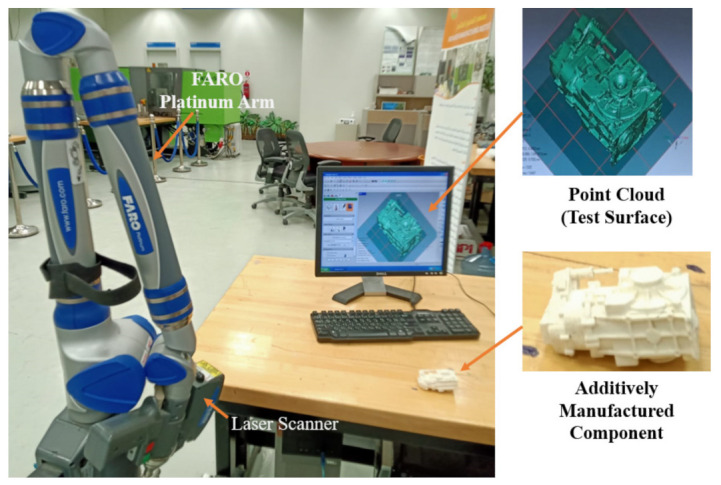
Set up to capture the shape of test specimens.

After the parts were fabricated, they were again checked for accuracy. Upon dismantling the supporting structures, the components are evaluated using 3D Comparison analysis for any deviations from the designed Computer-Aided Design (CAD) file. The 3D comparison method was used to accurately quantify the fabrication error on components that are produced at their optimal orientations. It is considered one of the most reliable and comprehensive ways for dynamically depicting surface differences between test surfaces and the reference CAD model [93]. This deviation assessment deploying a 3D comparison approach is carried out in Geomagics Control^®^ (Courtesy: 3D Systems). At the outset of this operation, the test model (manufactured component) must be set up on the reference CAD model deploying the best fit alignment. Following that, the analysis software approximates the best fit among the test and references objects automatically. The test and reference entities are in the identical coordinate system because of this best fit alignment. Additionally, in this research, the average maximum deviation in the outward direction is preferred as the statistic to quantify component quality. This statistic is useful since it exemplifies component variance, enabling the approximation of gap across the test specimens and the reference CAD model. As illustrated in Figure 12, the test specimens (components built in the best possible orientation) were gathered as a point cloud set, employing the laser scanner equipped on the Faro Platinum arm (FARO, Lake Mary, FL, USA).

**Figure 13:**
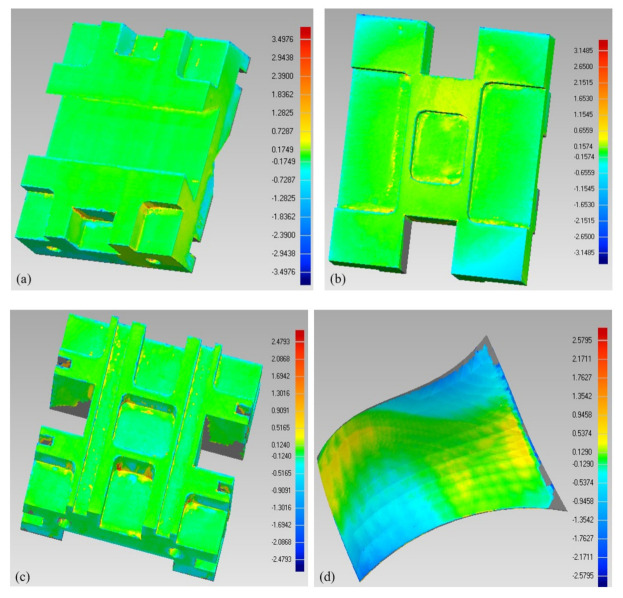

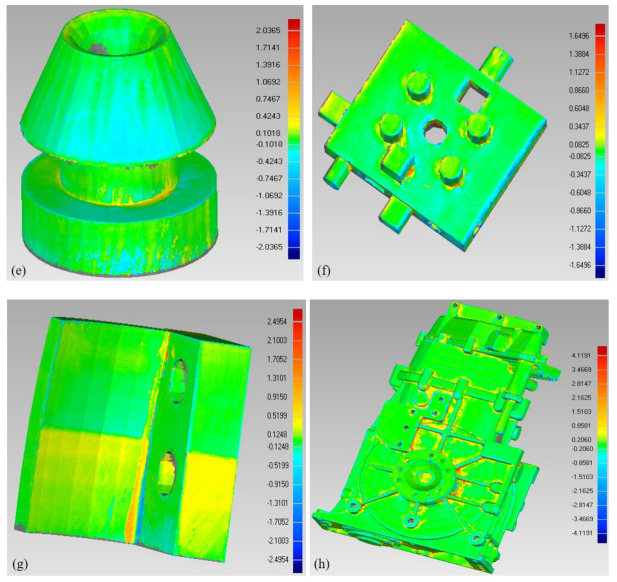

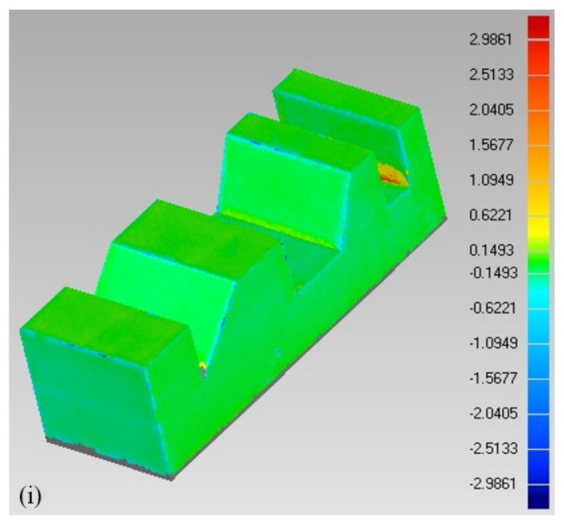
Deviation between the 3D printed parts and their CAD models from the 3D comparison—(**a**) Component 1; (**b**) Component 2; (**c**) Component 3; (**d**) Component 4; (**e**) Component 5; (**f**) Component 6; (**g**) Component 7; (**h**) Component 8; (**i**) Component 9.

The surface of the test specimens is scanned and loaded as an STL model into Geomagics control^®^. In order to inspect the test specimens with the reference CAD, the 3D comparison analysis was performed. The 3D comparison analysis reports the findings as an error scale (or deviation) depending on the shortest distance between the test model and the reference model’s surface. The deviations in the outside direction are considered to maintain consistency in the measurements. It implies that only positive deviations have been assessed. The positive deviation in this work represents the deviation between the CAD model (the reference model) and the 3D printed parts (the scanned test specimens) in the outward direction. This deviation is measured from the CAD model (outward direction) towards the surface of the test specimen. The outcomes of the 3D comparison study are graphically depicted in Figure 13a–i, representing the deviation between the 3D printed components fabricated at their optimum orientations and the reference CAD model. These deviations were measured in mm. There is a marginal difference in the range of 0.07–0.17 mm between the fabricated components and the CAD model, based on the findings (see Table 6). It also confirms that components manufactured in their optimal orientation have attained reasonable accuracy while also reducing build time and material consumption.

The maximum deviation in the manufactured components is well below 200 microns, which is extremely low, given the intricacy of the parts. This demonstrates how crucial it is to properly orient manufactured components in order to improve their quality while also keeping a close eye on the build time. It is important to strike the right balance between accuracy, build time, and component orientation. It is undoubtedly feasible that while certain component’s accuracy improves, build time increases as well. Because the study focuses on multiple components rather than just one, the overall build time is more important than the individual production time. Moreover, it is challenging to correlate the object orientation and production time because the objective is to find the ideal balance between accuracy, build time, and component orientation. Undoubtedly, there is a chance that, in some cases, the build time of the particular component can be hampered in order to preserve the component’s intended quality.

## 5. Conclusions

The multi-part production orientation optimization problem in AM was outlined and examined. It is a variation of the classic multi-objective traveling salesman problem, which is NP-hard. This research proposes a novel technique for multi-part AM production that reduces fabrication time and material usage, hence conserving energy. The technique entails using GA in the MATLAB environment to optimize an objective function based on two criteria (accuracy and time). The optimal building orientation seeks to shorten the production time and enhance the component’s quality. The most significant contribution of this research is to aid the decision-makers in determining the optimal orientations for multiple components in AM. A case study was carried out with nine parts of various shapes, sizes, and complexity. The proposed scheme has a number of advantages in comparison to the existing approaches. The new method has taken accuracy into account in addition to build time, whereas the existing approaches focused only on minimizing build time. It also considered complexity as one of the main goals that was lacking in earlier studies. The suggested method can therefore be applied to any complex component and any AM machine. In addition to figuring out the best orientation for multi-AM production, it has also validated the accuracy of the manufactured parts.

When compared to the traditional procedure, where many parts are manufactured in their random orientations, the proposed strategy saves 2 h of fabrication time and 10 cm^3^ of support material. The effectiveness of the suggested approach is also quantified by employing 3D comparison analyses to estimate the accuracy of manufactured components. This demonstrates that the embraced technique is a promising strategy that AM users or decision-makers can explore when determining the best orientations for producing numerous components in a single build. However, more research is needed to develop more appropriate global objectives and investigate different optimization methods in order to refine the proposed approach. In the future, the proposed technique should be adopted for multi-part manufacturing in alternative AM processes and different materials, such as powder-based AM, to reduce fabrication time and material consumption. This technique should also be evaluated with more components and a higher level of complexity. Several other GA operators should be tested, and their values should be varied to further increase GA’s performance.

## Figures and Tables

**Table 1:** Optimization approaches adopted to achieve the best build orientation.

Author	Year	Adopted Techniques	Optimization Objectives
[41]	1995	Weighted Sum Function (WSF)	Part Accuracy (PA); Build Time (BT)
[42]	1997	Self-Developed Algorithm (SDA)	Surface Quality (SQ); BT; Support Structure Volume (SS)
[43]	1998	SDA	Cost (CC); SS; Contact Area with Support
[44]	1998	SDA	BT; PA; SQ
[45]	1998	GA	PA; BT; SS
[15]	1999	SDA	CC; BT; PA; SQ
[46]	2001	GA	BT; SQ
[47]	2003	GA	Volumetric Error (VE)
[48]	2004	GA	SQ; BT
[49]	2004	GA	SQ; BT
[50]	2005	GA	Post-Processing Time (PPT) and CC
[51]	2007	GA	PPT
[52]	2009	GA	BT; SQ; PPT
[53]	2011	GA and Particle Swarm Algorithm (PSO)	SQ; BT
[54]	2011	GA	SQ; Energy Consumption
[55]	2013	GA	VE
[56]	2015	GA	Cylindricity and Flatness Errors; SS
[57]	2015	SDA	SS
[58]	2016	Principal Component Analysis	VE
[59]	2017	GA	BT; CC; Production Quality
[60]	2017	GA	SQ; BT; CC; Yield Strength; Tensile Strength
[27]	2017	GA	SS; Support Structure Accessibility
[61]	2018	Point Clustering Algorithm	Count of Material Changes
[62]	2018	WSF	GD&T Values; Production Time
[63]	2018	GA	Adaptive Feature Roughness; BT
[64]	2018	Hybrid PSO-BFO Algorithm	Hardness; Flexural Modulus; Tensile Strength; SQ
[65]	2019	Taguchi Method	SS; SQ
[66]	2020	Non-Stochastic Feature Recognition Approach	Staircase Effect
[67]	2021	Nesting Algorithm	CC and BT
[68]	2022	Mixed integer linear programming, GA, and PSO	Make span
[69]	2022	SDA	Material and Energy Consumption

**Table 2:** Explanation of orientation number.

Orientation	Description
1	Bottom face is the base surface and *θ* = 0°
2	Top face is the base surface and *θ* = 180°
3	Front face is the base surface and *θ* = 90° (XZ plane, rotated along *X*-axis, counterclockwise)
4	Rear face is the base surface and *θ* = 90° (XZ plane, rotated along *X*-axis, clockwise)
5	Right face is the base surface and θ = 90° (YZ plane, rotated along *Y*-axis, clockwise)
6	Left face is the base surface and *θ* = 90° (YZ plane, rotated along *Y*-axis, counterclockwise)

**Table 3:** Example for roulette wheel selection.

Chromosomes		Objective Function Value (*Z*)	Fitness Function Value (*f*)	Probability of Each Chromosome (*p_i_*)	Cumulative Probability (*P_i_*)	Random Number	Selection
I	2 1 3 4 2 3	12.80	12.80	0.30	0.30	0.57	II
II	4 6 2 3 1 1	13.69	13.69	0.32	0.61	0.30	I
III	3 4 5 1 2 4	16.78	16.78	0.39	1.00	0.09	I
		Σ *f*	43.27				

**Table 4:** Details of nine selected components.

Component	Description	Dimension (mm)
Component 1	Prismatic part and features. Fifty-seven faces, along with their loops, edges, vertices, face orientation, face type, and face area. Fourteen manufacturing features.	70 × 20 × 55
Component 2	Prismatic part and features. Seventy-two faces, including their loops, edges, vertices, face direction, face type, and the face area. Twelve manufacturing features.	49.5 × 63 × 18
Component 3	Prismatic part and features. Eighty-nine faces, along with their loops, edges, vertices, face direction, face type, and the face area. Twenty-six manufacturing features.	49 × 49.5 × 16
Component 4	The freeform surface is a Non-Uniform Rational B-Spline (NURBS).	52.5 × 48 × 27.047
Component 5	Rotational part comprising different internal and external features (symmetric in nature).	27.988 × 27.988 × 40.882
Component 6	The prismatic part contains different features such as pocket through, pocket blind, hole through, and external cylinders.	33 × 32 × 10
Component 7	Combination of prismatic and freeform surfaces which include through holes on the left surface.	31.287 × 50 × 46.8
Component 8	The real part is a car gearbox (with four-speed and reverse transaxle).	82.44 × 51.588 × 43.484
Component 9	The prismatic part includes different features such as the staircase and step through.	20 × 60 × 20

**Table 5:** Computed values of *Q* and *T* for various components.

Orientation	1		2		3		4		5		6	
Criteria	*Q*	*T*	*Q*	*T*	*Q*	*T*	*Q*	*T*	*Q*	*T*	*Q*	*T*
Component 1	31.662	3.24	16.302	3.35	44.429	4.560	44.428	4.560	26.108	5.110	26.107	5.110
Component 2	52.623	2.21	34.512	2.28	29.140	3.320	29.130	3.320	29.743	4.070	29.744	4.070
Component 3	86.560	2.17	45.380	2.26	46.915	3.300	46.914	3.300	55.655	3.210	55.654	3.210
Component 4	1.400	1.34	−1.000	1.27	1.400	0.170	6.500	0.170	1.400	0.240	1.400	1.000
Component 5	5.208	1.21	0.973	1.34	−1.506	1.520	−1.506	1.520	−1.506	1.520	−1.506	1.520
Component 6	12.114	0.35	7.126	0.46	11.118	1.060	11.582	1.110	10.780	1.150	11.708	1.120
Component 7	4.553	3.08	−8.811	3.12	−9.855	3.090	−8.438	3.170	6.401	3.060	6.439	2.590
Component 8	4.200	9.31	4.100	9.29	4.000	8.050	3.875	8.040	4.500	8.570	4.575	9.360
Component 9	4.140	2.16	4.140	2.10	3.646	1.370	6.547	1.170	7.403	1.200	7.403	1.200

**Table 6:** Measured deviations of components post-fabrication.

Components	1	2	3	4	5	6	7	8	9
Average Maximum Deviation (mm)	0.15	0.11	0.10	0.17	0.09	0.13	0.13	0.16	0.07

## Data Availability

The data presented in this study are available in the article.

## References

[1] Dalpadulo E., Pini F., Leali F. (2020). Integrated CAD Platform Approach for Design for Additive Manufacturing of High Performance Automotive Components. Int. J. Interact. Des. Manuf..

[2] Gao W., Zhang Y., Ramanujan D., Ramani K., Chen Y., Williams C.B., Wang C.C.L., Shin Y.C., Zhang S., Zavattieri P.D. (2015). The Status, Challenges, and Future of Additive Manufacturing in Engineering. Comput.-Aided Des..

[3] Thompson M.K., Moroni G., Vaneker T., Fadel G., Campbell R.I., Gibson I., Bernard A., Schulz J., Graf P., Ahuja B. (2016). Design for Additive Manufacturing: Trends, Opportunities, Considerations, and Constraints. CIRP Ann..

[4] Ford S., Despeisse M. (2016). Additive Manufacturing and Sustainability: An Exploratory Study of the Advantages and Challenges. J. Clean. Prod..

[5] Gibson I., Rosen D., Stucker B., Khorasani M. (2021). Materials for Additive Manufacturing. Additive Manufacturing Technologies.

[6] Foresti R., Ghezzi B., Vettori M., Bergonzi L., Attolino S., Rossi S., Tarabella G., Vurro D., von Zeppelin D., Iannotta S. (2021). 3D Printed Masks for Powders and Viruses Safety Protection Using Food Grade Polymers: Empirical Tests. Polymers.

[7] Rezvani Ghomi E., Khosravi F., Saedi Ardahaei A., Dai Y., Neisiany R.E., Foroughi F., Wu M., Das O., Ramakrishna S. (2021). The Life Cycle Assessment for Polylactic Acid (PLA) to Make It a Low-Carbon Material. Polymers.

[8] Oliveira J.P., LaLonde A.D., Ma J. (2020). Processing Parameters in Laser Powder Bed Fusion Metal Additive Manufacturing. Mater. Des..

[9] Lacroix R., Seifert R.W., Timonina-Farkas A. (2020). Benefiting from Additive Manufacturing for Mass Customization across the Product Life Cycle.

[10] Lacroix R., Timonina-Farkas A., Seifert R.W. (2020). Utilizing Additive Manufacturing and Mass Customization under Capacity Constraints.

[11] Zhang Y., Bernard A. (2014). Grouping Parts for Multiple Parts Production in Additive Manufacturing. Procedia CIRP.

[12] Matos M.A., Rocha A.M.A.C., Costa L.A. (2021). Many-Objective Optimization of Build Part Orientation in Additive Manufacturing. Int. J. Adv. Manuf. Technol..

[13] Spoerk M., Holzer C., Gonzalez-Gutierrez J. (2020). Material Extrusion-Based Additive Manufacturing of Polypropylene: A Review on How to Improve Dimensional Inaccuracy and Warpage. J. Appl. Polym. Sci..

[14] Vahabli E., Rahmati S. (2017). Hybrid Estimation of Surface Roughness Distribution in FDM Parts Using Analytical Modeling and Empirical Investigation. Int. J. Adv. Manuf. Technol..

[15] Xu F., Loh H.T., Wong Y.S. (1999). Considerations and Selection of Optimal Orientation for Different Rapid Prototyping Systems. Rapid Prototyp. J..

[16] Byun H.S., Lee K.H. (2005). Determination of Optimal Build Direction in Rapid Prototyping with Variable Slicing. Int. J. Adv. Manuf. Technol..

[17] Li A., Zhang Z., Wang D., Yang J. Optimization Method to Fabrication Orientation of Parts in Fused Deposition Modeling Rapid Prototyping. Proceedings of the 2010 International Conference on Mechanic Automation and Control Engineering.

[18] Wang W.M., Zanni C., Kobbelt L. (2016). Improved Surface Quality in 3D Printing by Optimizing the Printing Direction. Comput. Graph. Forum.

[19] Zhang Y., De Backer W., Harik R., Bernard A. (2016). Build Orientation Determination for Multi-Material Deposition Additive Manufacturing with Continuous Fibers. Procedia CIRP.

[20] Luo Z., Yang F., Dong G., Tang Y., Zhao Y.F. (2016). Orientation Optimization in Layer-Based Additive Manufacturing Process.

[21] Wang C. (2022). Simultaneous Optimization of Build Orientation and Topology for Self-Supported Enclosed Voids in Additive Manufacturing. Comput. Methods Appl. Mech. Eng..

[22] Nezhadfar P.D., Thompson S., Saharan A., Phan N., Shamsaei N. (2021). Structural Integrity of Additively Manufactured Aluminum Alloys: Effects of Build Orientation on Microstructure, Porosity, and Fatigue Behavior. Addit. Manuf..

[23] Zhou Y., Nomura T., Saitou K. (2020). Anisotropic Multicomponent Topology Optimization for Additive Manufacturing With Build Orientation Design and Stress-Constrained Interfaces. J. Comput. Inf. Sci. Eng..

[24] Barclift M., Armstrong A., Simpson T.W., Joshi S.B. (2017). CAD-Integrated Cost Estimation and Build Orientation Optimization to Support Design for Metal Additive Manufacturing.

[25] Langelaar M. (2018). Combined Optimization of Part Topology, Support Structure Layout and Build Orientation for Additive Manufacturing. Struct. Multidiscip. Optim..

[26] Baturynska I. (2018). Statistical Analysis of Dimensional Accuracy in Additive Manufacturing Considering STL Model Properties. Int. J. Adv. Manuf. Technol..

[27] Chowdhury S., Mhapsekar K., Anand S. (2017). Part Build Orientation Optimization and Neural Network-Based Geometry Compensation for Additive Manufacturing Process. J. Manuf. Sci. Eng..

[28] Qin Y., Qi Q., Scott P.J., Jiang X. (2019). Determination of Optimal Build Orientation for Additive Manufacturing Using Muirhead Mean and Prioritised Average Operators. J. Intell. Manuf..

[29] Ransikarbum K., Pitakaso R., Kim N. (2019). Evaluation of Assembly Part Build Orientation in Additive Manufacturing Environment Using Data Envelopment Analysis. MATEC Web Conf..

[30] Cheng L., To A. (2019). Part-Scale Build Orientation Optimization for Minimizing Residual Stress and Support Volume for Metal Additive Manufacturing: Theory and Experimental Validation. Comput.-Aided Des..

[31] Wang C., Qian X. (2020). Simultaneous Optimization of Build Orientation and Topology for Additive Manufacturing. Addit. Manuf..

[32] Zhang Y., Harik R., Fadel G., Bernard A. (2018). A Statistical Method for Build Orientation Determination in Additive Manufacturing. Rapid Prototyp. J..

[33] Das P., Chandran R., Samant R., Anand S. (2015). Optimum Part Build Orientation in Additive Manufacturing for Minimizing Part Errors and Support Structures. Procedia Manuf..

[34] Zwier M.P., Wits W.W. (2016). Design for Additive Manufacturing: Automated Build Orientation Selection and Optimization. Procedia CIRP.

[35] Delfs P., Töows M., Schmid H.-J. (2016). Optimized Build Orientation of Additive Manufactured Parts for Improved Surface Quality and Build Time. Addit. Manuf..

[36] Jaiswal P., Patel J., Rai R. (2018). Build Orientation Optimization for Additive Manufacturing of Functionally Graded Material Objects. Int. J. Adv. Manuf. Technol..

[37] Khodaygan S., Golmohammadi A.H. (2018). Multi-Criteria Optimization of the Part Build Orientation (PBO) through a Combined Meta-Modeling/NSGAII/TOPSIS Method for Additive Manufacturing Processes. Int. J. Interact. Des. Manuf..

[38] Pereira S., Vaz A.I.F., Vicente L.N. (2018). On the Optimal Object Orientation in Additive Manufacturing. Int. J. Adv. Manuf. Technol..

[39] Vaz A.I.F., Vicente L.N. (2007). A Particle Swarm Pattern Search Method for Bound Constrained Global Optimization. J. Glob. Optim..

[40] Vaz A.I.F., Vicente L.N. (2009). PSwarm: A Hybrid Solver for Linearly Constrained Global Derivative-Free Optimization. Optim. Methods Softw..

[41] Cheng W., Fuh J.Y.H., Nee A.Y.C., Wong Y.S., Loh H.T., Miyazawa T. (1995). Multi-objective Optimization of Part- Building Orientation in Stereolithography. Rapid Prototyp. J..

[42] Lan P.-T., Chou S.-Y., Chen L.-L., Gemmill D. (1997). Determining Fabrication Orientations for Rapid Prototyping with Stereolithography Apparatus. Comput.-Aided Des..

[43] Alexander P., Allen S., Dutta D. (1998). Part Orientation and Build Cost Determination in Layered Manufacturing. Comput.-Aided Des..

[44] McClurkin J.E., Rosen D.W. (1998). Computer-aided Build Style Decision Support for Stereolithography. Rapid Prototyp. J..

[45] Hur J., Lee K. (1998). The Development of a CAD Environment to Determine the Preferred Build-up Direction for Layered Manufacturing. Int. J. Adv. Manuf. Technol..

[46] Hur S.-M., Choi K.-H., Lee S.-H., Chang P.-K. (2001). Determination of Fabricating Orientation and Packing in SLS Process. J. Mater. Process. Technol..

[47] Masood S.H., Rattanawong W., Iovenitti P. (2003). A Generic Algorithm for a Best Part Orientation System for Complex Parts in Rapid Prototyping. J. Mater. Process. Technol..

[48] Thrimurthulu K., Pandey P.M., Venkata Reddy N. (2004). Optimum Part Deposition Orientation in Fused Deposition Modeling. Int. J. Mach. Tools Manuf..

[49] Pandey P.M., Thrimurthulu K., Reddy N.V. (2004). Optimal Part Deposition Orientation in FDM by Using a Multicriteria Genetic Algorithm. Int. J. Prod. Res..

[50] Kim H.-C., Lee S.-H. (2005). Reduction of Post-Processing for Stereolithography Systems by Fabrication-Direction Optimization. Comput.-Aided Des..

[51] Ahn D., Kim H., Lee S. (2007). Fabrication Direction Optimization to Minimize Post-Machining in Layered Manufacturing. Int. J. Mach. Tools Manuf..

[52] Canellidis V., Giannatsis J., Dedoussis V. (2009). Genetic-Algorithm-Based Multi-Objective Optimization of the Build Orientation in Stereolithography. Int. J. Adv. Manuf. Technol..

[53] Padhye N., Deb K. (2011). Multi-objective Optimisation and Multi-criteria Decision Making in SLS Using Evolutionary Approaches. Rapid Prototyp. J..

[54] Strano G., Hao L., Everson R.M., Evans K.E. (2011). Multiobjective Optimisation of Selective Laser Sintering Processes for Surface Quality and Energy Saving. Proc. Inst. Mech. Eng. Part B J. Eng. Manuf..

[55] Zhang J., Li Y. (2013). A Unit Sphere Discretization and Search Approach to Optimize Building Direction with Minimized Volumetric Error for Rapid Prototyping. Int. J. Adv. Manuf. Technol..

[56] Paul R., Anand S. (2015). Optimization of Layered Manufacturing Process for Reducing Form Errors with Minimal Support Structures. J. Manuf. Syst..

[57] Ezair B., Massarwi F., Elber G. (2015). Orientation Analysis of 3D Objects toward Minimal Support Volume in 3D-Printing. Comput. Graph..

[58] Luo N., Wang Q. (2016). Fast Slicing Orientation Determining and Optimizing Algorithm for Least Volumetric Error in Rapid Prototyping. Int. J. Adv. Manuf. Technol..

[59] Zhang Y., Bernard A., Harik R., Karunakaran K.P. (2017). Build Orientation Optimization for Multi-Part Production in Additive Manufacturing. J. Intell. Manuf..

[60] Brika S.E., Zhao Y.F., Brochu M., Mezzetta J. (2017). Multi-Objective Build Orientation Optimization for Powder Bed Fusion by Laser. J. Manuf. Sci. Eng..

[61] Mi S., Wu X., Zeng L. (2018). Optimal Build Orientation Based on Material Changes for FGM Parts. Int. J. Adv. Manuf. Technol..

[62] Al-Ahmari A.M., Abdulhameed O., Khan A.A. (2018). An Automatic and Optimal Selection of Parts Orientation in Additive Manufacturing. Rapid Prototyp. J..

[63] Huang R., Dai N., Li D., Cheng X., Liu H., Sun D. (2018). Parallel Non-Dominated Sorting Genetic Algorithm-II for Optimal Part Deposition Orientation in Additive Manufacturing Based on Functional Features. Proc. Inst. Mech. Eng. Part C J. Mech. Eng. Sci..

[64] Raju M., Gupta M.K., Bhanot N., Sharma V.S. (2019). A Hybrid PSO–BFO Evolutionary Algorithm for Optimization of Fused Deposition Modelling Process Parameters. J. Intell. Manuf..

[65] Golmohammadi A.H., Khodaygan S. (2019). A Framework for Multi-Objective Optimisation of 3D Part-Build Orientation with a Desired Angular Resolution in Additive Manufacturing Processes. Virtual Phys. Prototyp..

[66] Leirmo T.S., Martinsen K. (2020). Deterministic Part Orientation in Additive Manufacturing Using Feature Recognition. Procedia CIRP.

[67] Nesting Algorithm for Optimization Part Placement in Additive Manufacturing. https://www.researchsquare.com.

[68] Lee S.J., Kim B.S. (2022). Two-Stage Meta-Heuristic for Part-Packing and Build-Scheduling Problem in Parallel Additive Manufacturing. SSRN.

[69] Ma Z., Gao M., Guo K., Wang Q., Li L., Liu C., Zhu G., Liu Z. (2022). Analysis and Optimization of Energy Consumption for Multi-Part Printing Using Selective Laser Melting and Considering the Support Structure. Int. J. Precis. Eng. Manuf.-Green Technol..

[70] Jiang J., Xu X., Stringer J. (2019). Optimisation of Multi-Part Production in Additive Manufacturing for Reducing Support Waste. Virtual Phys. Prototyp..

[71] Jiang J., Xu X., Xiong Y., Tang Y., Dong G., Kim S. (2020). A Novel Strategy for Multi-Part Production in Additive Manufacturing. Int. J. Adv. Manuf. Technol..

[72] Makanda I.L.D., Yang M., Shi H., Guo W., Jiang P. (2022). A Multi-Part Production Planning System for a Distributed Network of 3D Printers under the Context of Social Manufacturing. Machines.

[73] Ransikarbum K., Ha S., Ma J., Kim N. (2017). Multi-Objective Optimization Analysis for Part-to-Printer Assignment in a Network of 3D Fused Deposition Modeling. J. Manuf. Syst..

[74] Bible T.E. PLA vs. ABS: Which Should I Use?. https://engineersbible.com/pla-vs-abs/.

[75] PLA vs. ABS: The Main Differences. https://all3dp.com/2/pla-vs-abs-filament-3d-printing/.

[76] Hay A. PLA vs. ABS: Which Filament Is Better for 3D Printing?. https://jiga.io/resource-center/3d-printing/pla-vs-abs-which-is-better-for-3d-printing/.

[77] HUBS 3D Printing with PLA vs. ABS: What’s the Difference?. https://www.hubs.com/knowledge-base/pla-vs-abs-whats-difference/.

[78] IEPASADMIN (2013). Using PLA for Long-Term Outdoor Applications.

[79] Zhang P., Liu J., To A.C. (2017). Role of Anisotropic Properties on Topology Optimization of Additive Manufactured Load Bearing Structures. Scr. Mater..

[80] Khan A.A., Nasr E.A., Ahmari A.A. (2015). An Independent CAPP System for Prismatic Parts. IJRAPIDM.

[81] Frank D., Fadel G. (1995). Expert System-Based Selection of the Preferred Direction of Build for Rapid Prototyping Processes. J. Intell. Manuf..

[82] Mian S.H., Al-Ahmari A. (2014). Enhance Performance of Inspection Process on Coordinate Measuring Machine. Measurement.

[83] Paul V., Ganeshkumar C., Jayakumar L. (2019). Performance Evaluation of Population Seeding Techniques of Permutation-Coded GA Traveling Salesman Problems Based Assessment: Performance Evaluation of Population Seeding Techniques of Permutation-Coded GA. IJAMC.

[84] Bhosale K.C., Pawar P.J. (2019). Material Flow Optimisation of Production Planning and Scheduling Problem in Flexible Manufacturing System by Real Coded Genetic Algorithm (RCGA). Flex. Serv. Manuf. J..

[85] Katoch S., Chauhan S.S., Kumar V. (2021). A Review on Genetic Algorithm: Past, Present, and Future. Multimed. Tools Appl..

[86] Wang H., Lin D., Li M.-Q. A Competitive Genetic Algorithm for Resource-Constrained Project Scheduling Problem. Proceedings of the 2005 International Conference on Machine Learning and Cybernetics.

[87] Andreica A., Chira C. (2015). Best-Order Crossover for Permutation-Based Evolutionary Algorithms. Appl. Intell..

[88] Rahmani Hosseinabadi A.A., Vahidi J., Saemi B., Sangaiah A.K., Elhoseny M. (2019). Extended Genetic Algorithm for Solving Open-Shop Scheduling Problem. Soft Comput..

[89] Xin J., Zhong J., Yang F., Cui Y., Sheng J. (2019). An Improved Genetic Algorithm for Path-Planning of Unmanned Surface Vehicle. Sensors.

[90] Gen M., Cheng R. (1997). Genetic Algorithms and Engineering Design.

[91] Jebari K., Madiafi M. (2013). Selection Methods for Genetic Algorithms. Int. J. Emerg. Sci..

[92] Yang X.-S., Chien S.F., Ting T.O., Yang X.-S., Chien S.F., Ting T.O. (2015). Chapter 1—Bio-Inspired Computation and Optimization: An Overview. Bio-Inspired Computation in Telecommunications.

[93] Hammad Mian S., Abdul Mannan M., Al-Ahmari A.M. (2014). The Influence of Surface Topology on the Quality of the Point Cloud Data Acquired with Laser Line Scanning Probe. Sens. Rev..

